# Changes in gram negative microorganisms’ resistance pattern during 4 years period in a referral teaching hospital; a surveillance study

**DOI:** 10.1186/2008-2231-20-28

**Published:** 2012-09-10

**Authors:** Hossein Khalili, Simin Dashti-Khavidaki, Mohammad-Reza Shahidi, Alireza Abdollahi, Sirous Jafari, Zahra Jahangard-Rafsanjani, Azita-Hajhosssein Talasaz

**Affiliations:** 1 Department of Clinical Pharmacy, Faculty of Pharmacy, Tehran University of Medical Sciences, Tehran, Iran; 2 Department of Pathology, Faculty of Medicine, Tehran University of Medical Sciences, Tehran, Iran; 3 Department of Infectious Diseases, Faculty of Medicine, Tehran University of Medical Sciences, Tehran, Iran

**Keywords:** Gram negative microorganism, Resistance pattern

## Abstract

**Background and purpose:**

Surveillance studies evaluating antimicrobial susceptibilities are of great value in preventing the spread of resistant pathogens by elucidating the trend of resistance in commonly used antibiotics and as a consequence providing information for prescribing the most appropriate agent. This study is a longitudinal antimicrobial resistance surveillance study designed to evaluate the trend in antimicrobial resistance to gram negative microorganisms from 2007 to 2010.

**Method:**

During a four-year period (2007–2010) isolates derived from all patients admitted to infectious diseases ward of Imam Khomeini Hospital, the major referral center for infectious disease in Iran with the highest admission rates, were evaluated. Based on disk diffusion method and zone of inhibition size, the microorganism was regarded as to be sensitive, resistant or has intermediate susceptibility to the antimicrobial agents.

**Results:**

The widest spread Gram-negative microorganism in all of isolates taken together in our study was *E.coli* (30%) followed by *Stenotrophomonas maltophilia* in 28.6% and *Enterobacter spp.* in 11.9%, respectively. The susceptibility to amikacin, imipenem, piperacillin/tazobactam, and nitrofurantoin was equal or above 50% for all microorganisms over four years. However, the susceptibility to ampicillin, ampicillin/sulbactam, cefotaxim, and ceftriaxone was less than 50% in derived isolates during the study period.

**Conclusion:**

In conclusion, the finding of the present study revealed that resistance rate to common antimicrobial agents in Iran is growing and isolates were susceptible mostly to broad-spectrum antibiotics including imipenem and piperacillin/tazobactam.

## Introduction

Antibiotic resistance is a critical challenge for infective diseases management around the word [1-3]. Infection with a resistant strain has been associated to higher rate of morbidity and mortality as well as prolonged length of hospital and intensive care unit stay and increased expenses for the healthcare systems [3,4].

Surveillance studies evaluating antimicrobial susceptibilities are of great value in providing information for prescribing the most appropriate agent [5]. These studies could be used as a guide in antimicrobial usage policies in order to halt the expansion of microorganism resistance [6-10].

Although there are few reports on the antibiotic resistance in Iran, many of them are simple point-in-time prevalence studies or evaluated pathogens involved in a specific infectious disease [11-14]. Present evaluation is a longitudinal antimicrobial resistance surveillance study designed to evaluate the trend in antimicrobial resistance to nosocomial origin gram negative microorganisms, from 2007 to 2010.

## Methods

During a four-year period (2007–2010) isolates biological samples from all patients admitted to Infectious Diseases Ward of Imam Khomeini Hospital Complex, the major referral center for infectious disease in Iran with the highest admission rates, were evaluated. The Institutional Review Board (IRB) and the Medical Ethics Committee of the hospital approved the study.

Isolates were sent to a central laboratory of the hospital for identification and antimicrobial susceptibility testing by Kirby – Bauer disc diffusion method. Based on zone of inhibition size the microorganism was regarded as to be sensitive, resistant or intermediate susceptibility to the antimicrobial agent. Microorganisms’ susceptibilities were investigated against those antimicrobials of clinical utility for the treatment of infections caused by susceptible Gram- negative bacteria available in the hospital. β-lactams such as ampicillin and piperacillin from penicillin class; cephalosporins including cefixime, cefotaxim, ceftazidim, ceftriaxone, cefepime; and the carbapenems such as imipenem were among evaluated antimicrobials. Combinations of penicillins with β-lactamase inhibitors including piperacillin-tazobactam and ampicillin-sulbactam, fluroquinolones such as ciprofloxacin, aminoglycosides including amikacin and gentamicin, nitrofurantoin and cotrimoxazole were those studied for antimicrobial resistance pattern in this surveillance. The trend in this pattern over four years was reviewed and reported.

## Results

In a four-year period from 2007 to 2010, 1745 isolates were sent to the central library of the hospital, from those 983 (56.3%) were confirmed to be Gram-negative pathogens. The most frequent specimen sources were blood (46.2%) followed by the urine (27%), and wound (13.7. The most wide spread Gram-negative microorganism in all of isolates taken together in our study was *E.coli* (30%) followed by *Stenotrophomonas maltophilia* in 28.6% and *Enterobacter spp.* in 11.9%, respectively. The frequency of microorganisms in terms of the derived isolates is shown in Table 1.

**Table 1:** Frequency of Gram-negative microorganisms according to the origins of specimen resource

**Microorganism**	**Blood**	**Urine**	**Wound**	**Other**	**Total**
E.Coli	14	201	40	30	285
Stenotrophomonas sp.	278	0	1	2	281
Enterobacter	64	42	5	6	117
Pseudomona	21	25	25	13	84
Acinetobacter	29	22	10	13	74
Kelebsiela	18	32	6	8	64
Proteus	2	9	20	11	42
Citrobacter	3	22	6	5	36
Total	429	353	113	88	983

The overall susceptibility of the specimens to antimicrobial agents did not remain the same over four years. For some of antimicrobial agents, the percentage of susceptible microorganisms was increased including ciprofloxacin (44.2 to 68.4%), piperacillin(33.3 to 66.7%) and piperacillin/tazobactam (77.8 to 89.5%). In contrast the susceptibility of nosocomial pathogens was reduced against nitrofurantoin (75.9 to 56%) in a four-year period. The susceptibility of microorganisms against all other antimicrobial agents showed non-steady pattern. The susceptibility to amikacin, imipenem, piperacillin/tazobactam, and nitrofurantoin was equal or above 50% for microorganisms all over four years. However, the susceptibility to ampicillin, ampicillin/sulbactam, cefotaxim, and ceftriaxone was less than 50% in derived isolates during the study period. The resistance rate of microorganism to Cefepime was increased in 2007–2008 while the susceptibilities were enhanced in 2009–2010.Comparison of the trend of resistance pattern of isolated bacteria of nosocomial originas well as the rate of frequency of each pathogen was illustrated in Table 2. Regarding the microorganisms, the most frequent resistances were seen in *Acientobacter* followed by *Citrobacter* with 50–73.7% and 58.3-65.8% resistant isolates, respectively. In contrast, *Enterobcter* was the most susceptible microorganism with resistance rate of 25.6-29.5% in the study period. The switches in the percent of resistant microorganism were not the same for all of the pathogens (Table 3). Proteus resistance was decreased in four-year period from 43.9% to 6.7% against all of the antibiotics taken together. *Acinetobacter* resistance to ciprofloxacin was increased whereas *Enterobacter* resistance rate to Gentamicin and Ciprofloxacin and *E.coli* resistance to Ceftazidim was decreased in four-year period. The number of resistant *Entrobacter i*solates to Cefixim was increased. From 2008 to 2010 the susceptibility rate of *Acinetobacter* against Ampicillin/sulbactam was reduced.

**Table 2:** Comparison of resistance pattern of isolated bacteria of nosocomial origin in four-year period

**Antimicrobial agent/ microorganism**	**Susceptibility**											
	**Sensitive; n (%)**				**Intermediate; n (%)**				**Resistant; n (%)**			
	**2007**	**2008**	**2009**	**2010**	**2007**	**2008**	**2009**	**2010**	**2007**	**2008**	**2009**	**2010**
Amikacin												
*Acinetobacter*	8	13	7	1	1	0	0	1	9	6	9	8
*Citrobacter*	4	5	11	N/A	1	0	0	N/	7	7	20	N/
*E.Coli*	62	85	53	26	2	0	0	A	10	8	16	A
*Enterobacter*	20	34	25	13	0	0	1	0	1	1	3	4
*Kelebsiella*	13	10	12	4	2	0	0	0	7	1	6	1
*Proteus spp.*	8	4	13	5	0	0	0	1	3	0	1	1
*Pseudomonas sp.*	9	18	12	11	0	0	0	0	2	3	11	6
*Stenotrophomonas sp.*	9	40	83	25	2	1	0	0	11	31	29	24
Total	133 (69.6%)	209 (78.3%)	216 (69.2%)	85 (64.4%)	8 (4.2%)	1 (0.3%)	1 (0.3%)	1 3 (2.3%)	50 (26.2%)	57 (21.3%)	95 (30.4%)	44 (33.3%)
Ampicillin/sulbactam												
*Acinetobacter*	1	5	11	3	0	0	0	0	3	3	10	5
*E.Coli*	3	6	15	7	0	0	2	1	2	12	49	15
*Enterobacter*	5	3	9	4	0	0	1	0	0	5	15	1
*Kelebsiella*	0	1	5	1	0	0	0	0	3	1	11	4
*Proteus spp.*	1	2	11	5	0	0	0	0	0	0	4	0
*Pseudomonas sp.*	0	0	3	0	0	0	0	0	2	4	20	14
*Stenotrophomonas sp.*	1	1	1	1	1	0	0	1	9	19	5	2
Total	11 (35.5%)	18 (29%)	55 (32%)	21 (32.8%)	1 (3.2%)	0 (0)	3 (1.7%)	2 (3.1%)	19 (61.3%)	44 (71%)	114 (66.3%)	41 (64.1%)
Ampicillin												
*E.Coli*	0	0	1	1	0	0	0	0	2	22	25	0
*Enterobacter*	0	0	2	0	0	0	0	0	1	5	4	2
Total	0 (0)	0 (0)	3 (9.4%)	1 (33.3%)	0 (0)	0 (0)	0 (0)	0 (0)	3 (100%)	27 (100%)	29 (90.6%)	2 (66.7%)
Cotrimoxazole												
*Acinetobacter*	7	6	5	1	0	0	0	0	14	10	13	8
*Citrobacter*	1	2	3	1	0	0	0	0	6	6	13	1
*E.Coli*	21	19	24	8	0	0	1	0	49	59	47	20
*Enterobacter*	12	24	21	8	0	0	0	0	11	13	7	4
*Kelebsiella*	6	5	7	2	0	0	0	0	17	3	12	4
*Proteus spp.*	4	2	4	3	0	1	0	0	7	3	9	1
*Pseudomonas sp.*	0	7	4	2	0	0	0	0	13	11	20	14
*Stenotrophomonas sp.*	22	78	107	35	0	0	0	0	1	2	2	10
Total	73 (38.2%)	143 (57%)	173 (58.2%)	60 (49.2%)	0 (0)	1 (0.4%)	1 (0.4%)	0 (0)	118 (61.8%)	107 (42.6%)	123 (41.4%)	62 (51.8%)
Cefepim												
*E.Coli*	3	4	7	2	0	0	0	0	3	3	5	1
*Enterobacter*	0	4	5	1	0	0	0	0	1	6	2	1
*Proteus spp.*	1	0	4	1	0	1	0	0	1	2	0	0
*Pseudomonas sp.*	0	0	2	1	0	0	0	0	1	8	4	3
Total	4 (40%)	8 (28.6%)	18 (62.1%)	5 (50%)	0 (0)	1 (3.6%)	0 (0)	0 (0)	6 (60%)	19 (67.9%)	11 (37.9%)	5 (50%)
Cefixime												
*Acinetobacter*	0	0	1	0	0	0	0	0	4	7	3	1
*E.Coli*	3	2	2	0	0	0	0	0	11	10	0	1
*Enterobacter*	3	6	1	0	1	2	0	1	3	11	2	4
*Proteus spp.*	1	0	4	1	0	1	0	0	1	2	0	0
*Pseudomonas sp.*	0	0	2	1	0	0	0	0	1	8	4	3
Total	7 (25%)	8 (40%)	10 (52.6%)	2 (16.7%)	1 (3.6%)	3 (15%)	0 (0)	1 (8.3%)	20 (71.4%)	9 (45%)	9 (47.4%)	9 (75%)
Cefotaxim												
*Acinetobacter*	1	1	0	1	0	0	0	0	4	3	5	1
*Citrobacter*	0	1	0	1	0	0	0	0	5	1	2	1
*Pseudomonas sp.*	0	1	0	1	0	0	0	0	4	1	4	1
*Stenotrophomonas sp.*	1	0	0	0	2	0	0	0	5	8	3	1
Total	2 (10%)	3 (18.7%)	0 (0)	3 (42.8%)	0 (0)	0 (0)	0 (0)	0 (0)	18 (90%)	13 (91.3%)	14 (100%)	4 (57.2%)
Ceftazidim												
*Acinetobacter*	0	0	2	2	0	0	0	0	4	8	5	2
*E.Coli*	3	3	7	15	0	0	1	0	4	16	43	6
*Enterobacter*	5	1	12	7	0	0	1	0	0	9	8	4
*Kelebsiella*	2	1	6	0	0	1	0	0	0	4	7	2
*Proteus spp.*	2	0	8	4	0	1	0	0	2	3	1	0
*Pseudomonas sp.*	2	0	4	3	0	0	0	0	1	12	12	4
*Stenotrophomonas sp.*	0	5	1	2	0	1	0	0	9	31	1	6
Total	14 (41.2%)	10 (10.4%)	40 (33.6%)	33 (57.9%)	0 (0)	3 (3.1%)	2 (1.7%)	0 (0)	20 (58.8%)	83 (86.4%)	77 (64.7%)	24 (42.1%)
Ciprofloxacin												
*Acinetobacter*	9	4	7	1	0	1	0	0	8	8	13	6
*E.Coli*	19	26	24	13	0	0	0	0	44	51	49	18
*Enterobacter*	12	18	24	8	0	0	0	0	8	7	8	2
*Kelebsiella*	6	4	10	4	2	0	0	0	20	7	20	7
*Proteus spp.*	4	2	8	6	0	0	0	0	5	3	7	0
*Pseudomonas sp.*	8	6	10	12	0	1	1	0	2	9	14	3
*Stenotrophomonas sp.*	15	39	109	45	0	0	0	0	3	0	0	1
Total	73 (44.2%)	99 (53.2%)	192 (63.2%)	80 (68.4%)	2 (1.2%)	2 (1.1%)	1 (0.3%)	0 (0)	90 (54.5%)	85 (45.7%)	111 (36.5%)	37 (31.6%)
Gentamicin												
*Acinetobacter*	5	9	4	1	1	0	0	0	9	2	6	5
*E.Coli*	18	49	24	12	1	1	2	0	32	35	36	8
*Enterobacter*	9	23	21	5	0	0	1	0	4	6	5	1
*Kelebsiella*	7	4	10	3	0	0	0	0	11	5	5	3
*Proteus spp.*	2	5	8	1	1	0	0	0	4	0	0	1
*Pseudomonas sp.*	5	12	1	4	0	0	0	0	3	7	10	2
*Stenotrophomonas sp.*	8	31	3	2	1	2	0	0	4	9	3	0
Total	54 (43%)	133 (66.5%)	71 (51.1%)	28 (58.3%)	4 (3%)	3 (1.5%)	3 (2.2%)	0 (0%)	67 (54%)	64 (32%)	65 (46.8%)	20 (51.7%)
Imipenem												
*Acinetobacter*	8	15	20	7	0	3	0	1	4	0	1	3
*E.Coli*	25	47	77	29	0	0	0	0	0	0	0	2
*Enterobacter*	12	30	31	13	0	0	0	0	0	0	0	1
*Kelebsiella*	10	8	19	8	0	0	0	0	0	0	0	0
*Proteus spp.*	7	6	17	6	0	0	0	0	0	0	0	0
*Pseudomonas sp.*	9	22	26	15	0	0	0	0	1	0	1	1
*Stenotrophomona s sp.*	2	10	2	2	0	1	1	0	20	49	7	9
Total	73 (74.5%)	132 (71.3%)	192 (95%)	80 (82.5%)	0 (0)	4 (2.2%)	1 (0.5%)	1 (1%)	25 (25.5%)	49 (26.5%)	9 (4.5%)	16 (16.5%)
Nitrofurantoin												
*Acinetobacter*	2	2	1	0	0	0	0	0	5	3	0	2
*E.Coli*	31	68	39	10	1	1	0	0	2	11	11	1
*Enterobacter*	4	8	8	1	1	1	0	0	1	8	3	0
*Kelebsiella*	5	2	5	3	0	0	1	0	1	3	3	2
*Proteus spp.*	1	1	0	0	0	0	0	0	2	0	1	1
*Pseudomonas sp.*	1	0	0	0	0	0	1	0	1	8	3	5
Total	44 (75.9%)	81 (69.8%)	53 (69.7%)	14 (56%)	2 (3.5%)	2 (1.7%)	2 (2.6%)	0 (0)	12 (20.7%)	33 (28.5%)	21 (27.6%)	11 (44%)
Piperacillin												
*Acinetobacter*	2	4	N/A	0	1	0	N/A	0	5	3	N/A	3
*E.Coli*	2	0	N/A	1	0	0	N/A	0	18	4	N/A	0
*Enterobacter*	9	14	N/A	6	0	0	N/A	0	2	4	N/A	1
*Kelebsiella*	3	2	N/A	1	0	0	N/A	0	6	4	N/A	0
Total	16 (33.3%)	20 (57.1%)	N/A	8 (66.7%)	1 (2.1%)	0 (0)	N/A	0 (0)	31 (64.6%)	15 (42.9%)	N/A	4 (33.3%)
Ceftriaxone												
*Acinetobacter*	1	6	4	0	0	0	0	0	15	13	10	9
*E.Coli*	17	40	16	13	0	1	1	0	51	50	55	17
*Enterobacter*	9	19	19	10	0	0	1	0	9	12	8	5
*Kelebsiella*	3	3	5	2	1	0	0	0	16	6	13	4
*Proteus spp.*	5	3	14	6	0	0	0	0	5	0	2	0
*Pseudomonas sp.*	2	2	4	5	0	4	1	0	7	13	19	8
*Stenotrophomonas sp.*	1	5	3	2	0	0	1	0	16	65	91	49
Total	39 (24.5%)	78 (32.2%)	65 (24.3%)	38 (29.2%)	1 (0.6%)	5 (2.1%)	4 (1.5%)	0 (0)	119 (74.8%)	159 (65.7%)	198 (74.2%)	92 (70.8%)
Piperacillin/tazobactam												
*Acinetobacter*	2	1	12	3	0	0	0	0	1	1	5	0
*E.Coli*	1	3	22	18	0	0	0	0	0	1	0	0
*Proteus spp.*	1	1	12	5	0	0	0	0	0	0	0	3
*Pseudomonas sp.*	1	3	13	6	0	0	0	1	1	0	4	0
*Stenotrophomonas sp.*	2	3	5	2	0	0	0	0	0	1	1	0
Total	7 (77.8%)	11 (78.6%)	64 (86.5%)	34 (89.5%)	0 (0)	0 (0)	0 (0)	1 (2.6%)	2 (22.2%)	3 (21.4%)	10 (13.5%)	3 (7.9%)

**Table 3:** Comparison of resistance pattern of isolated bacteria against different antimicrobial agents in four-year period

**Microorganism / Antimicrobial agent**	**Susceptibility**											
	**Sensitive; n (%)**				**Intermediate; n (%)**				**Resistant; n (%)**			
	**2007**	**2008**	**2009**	**2010**	**2007**	**2008**	**2009**	**2010**	**2007**	**2008**	**2009**	**2010**
Acinetobacter												
*Amikacin*	8	13	7	1	1	0	0	1	9	6	9	8
*Ampicillin/sulbactam*	1	5	11	3	0	0	0	0	4	8	21	8
*Cotrimoxazole*	7	6	5	1	0	0	0	0	14	10	13	8
*Cefixime*	0	0	1	0	0	0	0	0	4	7	3	1
*Cefotaxime*	1	1	0	1	0	0	0	0	4	3	5	1
*Ceftazidim*	0	2	2	0	0	0	0	0	4	10	7	2
*Ciprofloxacin*	9	4	7	1	0	1	0	0	8	8	13	6
*Gentamicin*	5	9	4	1	1	0	0	0	9	2	6	5
*Imipenem*	8	15	20	7	0	3	0	1	4	0	1	3
*Nitrofurantoine*	2	2	1	0	0	0	0	0	5	3	0	2
*Piperacillin*	2	4	N/A	0	1	0	N/A	0	5	3	N/A	3
*Ceftriaxone*	1	6	4	0	0	0	0	0	15	13	10	9
*Piperacillin/ tazobactam*	2	1	12	3	0	0	0	0	1	1	5	0
Total	46 (34.1%)	68 (45.9%)	74 (44.3%)	18 (23.7%)	3 (2.2%)	4 (2.7%)	0 (0)	2 (2.6%)	86 (63.7%)	74 (50%)	93 (55.7%)	56 (73.7%)
Citrobacter												
*Amikacin*	4	5	11	N/A	1	0	0	N/A	2	2	9	N/A
*Cotrimoxazole*	1	2	3	N/A	0	0	0		6	6	13	N/A
*Cefixime*	0	1	0	N/A	0	0	0	N/A	1	1	1	N/A
*Ciprofloxacin*	2	3	3	N/A	0	0	0		6	4	21	N/A
*Gentamicin*	1	5	4	N/A	0	0	0	N/A	5	1	11	N/A
*Imipenem*	4	3	20	N/A	0	0	0	N/A	0	0	0	N/A
*Ceftriaxone*	0	1	3	N/A	0	0	1	N/A	5	14	16	N/A
Total	12 (31.6%)	20 (41.7%)	44 (37.9%)	N/A	1 (2.6%)	0 (0)	1 (0.9%)	N/A	25 (65.8%)	28 (58.3%)	71 (61.2%)	N/A
E. coli												
*Amikacin*	62	85	53	26	2	0	0	0	10	8	16	4
*Ampicillin/sulbactam*	3	6	15	7	0	0	2	1	2	12	49	15
*Ampicillin*	0	0	1	1	0	0	0	0	2	22	26	1
*Cotrimoxazole*	21	19	24	8	0	0	1	0	49	59	47	20
*Cefepime*	3	4	7	2	0	0	0	0	3	23	5	1
*Cefixime*	3	2	2	0	0	0	0	0	11	10	0	1
*Ceftazidim*	3	3	7	15	0	0	1	0	4	16	43	6
*Ciprofloxacin*	19	26	24	13	0	0	0	0	44	51	49	18
*Gentamicin*	18	49	24	12	1	1	2	0	32	35	26	8
*Imipenem*	25	47	77	29	0	0	0	0	0	0	0	2
*Nitrofurantoine*	31	68	39	10	1	1	0	0	2	11	11	1
*Ceftriaxone*	17	40	16	13	0	1	1	0	51	50	55	17
*Piperacillin/ tazobactam*	1	3	22	18	0	1	1	0	0	1	0	0
Total	206 (49.1%)	352 (53.8%)	311 (48.1%)	154 (61.8%)	4 (0.9%)	4 (0.6%)	8 (1.2%)	1 (0.4%)	210 (50%)	298 (45.6%)	327 (50.6%)	94 (37.7%)
Enterobacter	20	34	25	13	0	0	1	0	1	1	3	1
*Amikacin*	5	3	9	4	0	0	1	0	0	5	15	1
*Ampicillin/sulbactam*	0	0	2	0	0	0	0	0	1	5	4	2
*Ampicillin*	12	24	21	8	0	0	0	0	11	13	7	4
*Cotrimoxazole*	0	4	5	1	0	0	0	0	1	6	2	1
*Cefepime*	3	6	1	0	1	2	0	1	3	11	2	4
*Cefixime*	5	11	12	7	0	0	1	0	0	9	8	4
*Ceftazidim*	12	18	24	8	0	0	0	0	8	7	8	2
*Ciprofloxacin*	9	23	21	5	0	0	1	0	4	6	5	1
*Gentamicin*	12	30	31	13	0	0	0	0	0	0	0	1
*Imipenem*	4	8	8	1	1	1	0	0	1	8	3	0
*Nitrofurantoine*	9	19	19	10	0	0	1	0	9	12	8	5
*Ceftriaxone*												
Total	91 (68.9%)	180 (67.7%)	178 (72.3%)	70 (72.2%)	2 (1.5%)	3 (1.1%)	5 (20.3%)	1 (0.1%)	39 (29.5%)	83 (31.2%)	63 (25.6%)	26 (26.8%)
Kelebsiella												
*Amikacin*	13	10	12	4	2	0	0	1	7	1	6	1
*Ampicillin/sulbactam*	0	1	5	1	0	0	0	0	3	1	11	4
*Cotrimoxazole*	6	5	7	2	0	0	0	0	17	3	12	4
*Cefixime*	1	3	1	0	0	0	0	0	9	3	0	1
*Ceftazidim*	2	1	6	0	0	1	0	0	0	4	7	2
*Ciprofloxacin*	6	4	10	4	2	0	0	0	12	3	10	3
*Gentamicin*	7	4	10	3	0	0	0	0	11	5	5	3
*Imipenem*	10	8	19	8	0	0	0	0	0	0	0	0
*Nitrofurantoine*	5	2	5	3	0	0	1	0	1	3	3	2
*Ceftriaxone*	3	3	5	2	1	0	0	0	16	6	13	4
Total	53 (39.5%)	41 (57.7%)	70 (50.7%)	27 (51.9%)	5 (3.7%)	1 (1.4%)	1 (0.7%)	1 (1.9%)	76 (56.7%)	29 (40.8%)	67 (48.5%)	24 (46.1%)
Proteus												
*Amikacin*	8	4	13	5	0	0	0	0	3	0	1	0
*Ampicillin/sulbactam*	1	2	11	5	0	0	0	0	0	0	4	0
*Cotrimoxazole*	4	2	4	3	0	1	0	0	7	3	9	1
*Cefepime*	1	0	4	1	0	1	0	0	1	2	0	0
*Ceftazidim*	2	0	8	4	0	1	0	0	2	3	1	0
*Ciprofloxacin*	4	2	8	6	0	0	0	0	5	3	7	0
*Gentamicin*	2	5	8	1	1	0	0	0	4	0	0	1
*Imipenem*	7	6	17	6	0	0	0	0	0	0	0	0
*Nitrofurantoine*	1	1	0	0	0	0	0	0	2	0	1	1
*Ceftriaxone*	5	3	14	6	0	0	0	0	5	0	2	0
*Piperacillin/ tazobactam*	1	1	12	5	0	0	0	0	0	0	0	0
Total	36 (54.5%)	26 (65%)	99 (79.8%)	42 (93.3%)	1 (1.5%)	3 (7.5%)	0 (0)	0 (0)	29 (43.9%)	11 (27.5%)	25 (20.2%)	3 (6.7%)
Pseudomona												
*Amikacin*	9	18	12	11	0	0	0	0	2	3	11	6
*Ampicillin/sulbactam*	0	0	3	0	0	0	0	0	2	4	20	14
*Cotrimoxazole*	0	7	4	2	0	0	0	0	13	11	20	14
*Cefepime*	0	0	2	1	0	0	0	0	1	8	4	3
*Ceftazidim*	2	0	4	3	0	0	0	0	1	12	12	4
*Ciprofloxacin*	8	6	10	12	0	1	1	0	2	9	14	3
*Gentamicin*	5	12	1	4	0	0	0	0	3	7	10	2
*Imipenem*	9	22	26	15	0	0	0	0	1	0	1	1
*Nitrofurantoine*	1	0	0	0	0	0	1	0	1	8	3	5
*Ceftriaxone*	2	2	4	5	0	4	1	0	7	13	19	8
*Piperacillin/ tazobactam*	1	3	13	6	0	0	0	1	1	0	4	3
Total	37 (52.1%)	70 (46.7%)	79 (39.9%)	59 (48%)	0 (0)	5 (3.3%)	3 (1.6%)	1 (0.8%)	34 (47.9%)	75 (50%)	118 (62.1%)	63 (51.2%)
Stenotrophomonas sp.												
*Amikacin*	9	40	83	25	2	1	0	1	11	31	29	24
*Ampicillin/sulbactam*	1	1	1	1	1	0	0	1	9	19	5	2
*Cotrimoxazole*	22	78	107	35	0	0	0	0	1	2	2	10
*Cefixime*	0	0	0	0	0	0	0	0	12	44	2	9
*Cefotaxime*	1	0	0	0	2	0	0	0	5	8	3	1
*Ceftazidim*	0	5	1	2	0	1	0	0	9	31	1	6
*Ciprofloxacin*	15	39	109	45	0	0	0	0	3	0	0	1
*Gentamicin*	8	31	3	2	1	2	0	0	4	9	3	0
*Imipenem*	2	10	2	2	1	1	0	0	20	49	7	9
*Ceftriaxone*	1	5	3	2	0	0	1	0	16	65	91	49
*Piperacillin/ tazobactam*	2	3	5	2	0	0	0	0	0	1	1	0
Total	61 (38.6%)	212 (44.5%)	314 (68.4%)	116 (50.6%)	7 (4.4%)	5 (1%)	1 (0.2%)	2 (0.9%)	90 (57%)	259 (54.5%)	144 (31.4%)	111 (48.5%)

## Discussion

Antimicrobial resistance is a widespread problem that health care providers are encountered with all over the world. Determining the specific pattern of antibiotic resistance especially in infectious diseases wards of main hospitals in every country is of great value for controlling the rate of increasing resistance as well as helping in empirical treatment. Since Imam Khomeini hospital Complex is the main center for infectious diseases in Iran with the highest admission rates, we can say that a serious problem of antimicrobial resistance to commonly used antibiotics exists among different isolates in Iran.

The most frequent isolated pathogen from all specimens taken together was *E. coli* followed by *S. maltophilia* and *Enterobacter sp*. with *E. coli* being the major derived pathogen from urine and *S. maltophilia* from bloodstream samples. In a similar Korean study, E. coli had the first rank in terms of the most prevalent organism isolated, *Pseudomona sp.,* and *Klebseilla sp.* had the next ranks among isolated gram-negative pathogens [15]. During 2000 to 2002, more than 220,000 isolated were collected from intensive care units of five countries including France, Germany, Italy, Canada and United States, in which the most common gram-negative pathogen was *E. coli* followed by *Pseudomona sp.*[16]. *S. maltophilia* which was the most common pathogen for bloodstream samples in the present study, were less common in western countries, however it was also seen frequently in Saudi isolates [17].

Antibiotic resistance among *Acinetobacter spp*., and *Citrobacter spp.* were more frequent in comparison with other isolated pathogens, and our susceptibility rates were similar to rates reported from other regions of the world [18,19]. However, the susceptibility rate of *Acinetobacter spp.* in our study was less than similar surveillance five-year study in Children Medical Center in Iran [20] and was more consistent with reports of antimicrobial resistance from other parts of the world [19,21]. Since that Iranian report dated back to approximately a decade ago, this may demonstrate the increase in the rate of *Acinetobacter* resistance in Iran like other countries over the world. Moreover, the resistance frequency rate of *Acinetobacter* to Ampicillin/sulbactam had an increasing trend after 2007. This was because the availability of generic Iranian formulation of this specific antibiotic in 2007 as well as the administration of oral dosage forms which was used widespread in that year.

The most commonly administered antibiotics in different countries all over the world are β-lactams, and decreased susceptibility of nosocomial pathogens to this therapeutic class of antibiotics has resulted in a major clinical disaster [22]. Iran is not an exception for this statement, and cephalosporines are commonly used in the country because of their availability as well as low rate of adverse events [12]. With respect to antibiotics, the most frequent resistance to antibiotics was observed in ampicillin (66.7-100%) and third generation cephalosporines consisting of cefotaxime (57.2-100%), ceftazidim (42.1-86.4%), cefixime (45-75%) and ceftriaxone (65.7-74.8%). To elucidate the importance of increasing resistance, it is worth mentioning that even the resistant rate of *Enterobacter* as the most susceptible microorganism to cefixime was increased in a four-year period. In contrast, the lowest resistant rates were seen with imipenem (4.5-26.5%),piperacillin/tazobactam (7.9-22.2%), and amikacin (21.3-33.3%). Resistance to third generation cephalosporines in this study was higher than similar studies evaluating the susceptibility rates of gram-negative pathogens [23-25]. This higher resistance can be attributed to the frequent use of third generation cephalosporines in the empirical management of infectious in Iran as the resistance rate of *E.coli* to ceftazidim was reduced after its administration was reduced and it was omitted from local protocols of empiric treatment for a period of time. Resistance of microorganisms to Cefepime, as a fourth generation cephalosporine, had an increasing trend in 2007–2008 but with restrictions in its usage the susceptibility rates were improved in 2009–2010.In contrast to previous studies that reported an increase in the resistance rate to fluoroquinolones [26,27], we found an improving susceptibility to ciprofloxacin in our study in a four-year period with *Acinetobacter* as an exception. This can be partly explained by the fact that fluoroquinolones are not routinely used as an empirical antibiotic for infectious diseases in Imam Hospital. In the present study, resistance to ciprofloxacin was 31.6-54.5% that was less than similar previous evaluation in Argentina with resistance rate of more than 80% [28]. On the other hand, the majority of the nosocomial pathogens from various specimen resources in the present study were susceptible to imipenem and this was consistent through the assessment period. As there was a correlation between previous use of fluoroquinolones and imipenem resistance [29,30], the low administration rate of ciprofloxacin and as a consequence improved sensitivity of organisms to this agent can be the reason behind the susceptibility of most of the organisms to imipenem. The low resistance rate of isolates to imipenem was also reported in previous Belgian and Polish studies with 13% and 8% resistant isolated, respectively [23,25]. In contrast, in Turkish patients resistance to imipenem was slightly more prevalent than that of our study [31]. Amikacin was among the most active antimicrobial agents against isolates with the low resistant rate of 21.3-33.3%. Studies performed over a long time period revealed that the increase in resistance to aminoglycosides is milder than for any other antimicrobial agent even with continued administration [32,33].

In conclusion, the finding of the present study revealed that resistance rate to common antimicrobial agents in Iran is growing and isolates were susceptible mostly to broad-spectrum antibiotics including imipenem and piperacillin/tazobactam. As the antibiotic resources of developing countries including Iran are limited, periodic surveillances of antimicrobial resistance patterns play a vital role in controlling the spread of resistant strains as well as implementing protocols for halting the process. Moreover, it is rational to establish a committee for appropriate antibiotic administration to control the use of antimicrobial agents at the same time of performing surveillance studies for the aim of effective infection management. Such surveillance studies could help in limiting the rate of antimicrobial resistance all over the world.

## Competing interests

The authors have not any conflict of interest about this work and have not any financial support.

## Authors’ contributions

MRS collected the culture and anti-biogram results of the patients from the microbiology laboratory. HK is main supervisor of the study and organized the data, SJ was responsible for clinical interpretation of the results, AA was responsible for the laboratory data analysis, Statistical analysis was done by ZJR and SDK and AH-T prepared the manuscript. All authors read and approved the final manuscript.
